# Phylogeography of a tough rock survivor in European dry grasslands

**DOI:** 10.1371/journal.pone.0179961

**Published:** 2017-06-22

**Authors:** Daniela Listl, Peter Poschlod, Christoph Reisch

**Affiliations:** Institute of Plant Sciences, University of Regensburg, Regensburg, Germany; National Cheng Kung University, TAIWAN

## Abstract

Phylogeographic analyses of plants in Europe have revealed common glacial refugia and migration routes for several trees and herbs with arctic-alpine distributions. The postglacial histories of dry grassland species in central Europe have rarely been analyzed, even though the extremely species-rich habitat is threatened. *Sedum album* (Crassulaceae) is a common inhabitant of rocky sites in central European dry grasslands. We inferred the phylogeographic history of *S*. *album* over its distribution range in Europe. Genetic diversity within and differentiation between 34 *S*. *album* populations was examined using AFLP markers. Population isolation was indicated based on the rarity of the fragments and by isolation-by-distance effects. We sequenced the trnL-trnF region in 32 populations and used chloroplast microsatellites to analyze chloroplast haplotype distributions. Two distinct *S*. *album* lineages were detected. One lineage was comprised of populations from eastern and central parts of central Europe, and the Apennine Peninsula. A second lineage was comprised of populations from the Iberian Peninsula and western and northern parts of central Europe. Glacial refugia were identified based on the accumulation of ancient chloroplast haplotypes, high diversity of AFLP fragments within populations, and high levels of rare fragments in Liguria, Serbia, the Apennine and Iberian peninsulas. Cryptic refugia were detected in the Czech Republic and Slovakia. Isolation by distance was present all over the distribution range, and it was separately detected in southwestern and central Europe. In western Europe, where a contact zone between the two lineages can be expected, no isolation by distance was detected. Our results suggest migration routes of *S*. *album* northeastward from glacial refugia in southern Iberia, northward from the Apennine Peninsula, and northward and westward from the southeastern parts of central Europe. Therefore, central European grasslands were recently colonized by northern cryptic populations and source populations originating in the east and the Apennine Peninsula.

## Introduction

The geographical distribution of plant species has always been influenced by climatic and edaphic factors. Quaternary ice ages dramatically reduced the habitats of most species [1–3]. Large parts of northern Europe, the Alps, and the Pyrenees were covered by ice, and steppe-tundra habitats with cold and dry conditions dominated the vegetation in most parts of western and central Europe. Most species were only able to survive in southern and eastern Europe where climatic conditions were milder than those in northern parts.

Nevertheless, next to the general contradiction-expansion theories, the plants and animals could have survived in northern areas characterized by suitable microclimate [4]. For many species, cryptic refugia in areas with sheltered topographies (e.g. river valleys and caves in river valleys) have been described in studies that are more recent [5–7].

Comparative phylogeographic studies identified patterns in the distribution of genetic diversity that could explain collective areas of glacial refugia and migration routes in Eurasian areas [3,8]. The Iberian Peninsula harbors the glacial refugia of several Eurasian species, and trees and shrubs from this region headed northward and westward after the last glacial maxima (LGM) [9–12]. Additional potential glacial refugia were identified for the genus *Helianthemum* in southern France and near the Pyrenees, from where the species migrated northward and westward [13]. Refugia were also suggested in Italy and on the Balkan Peninsula for several species [9,12,14–16], which expanded northward and westward to central Europe. Mountain ranges, particularly the Alps and the Pyrenees, form natural barriers to the migration routes of European plant species [11,17,18]. Rivers can form barriers and–more likely for most plants—serve as transportation routes for dispersal or as cryptic refugia [5,19].

In addition to archeobotanical findings (most notably pollen depositions but also macro-rests) genetic techniques are the methods of choice used to reveal the postglacial history of plant species [20]. Possible colonization scenarios are based on high haplotype diversity in glacial refugia. In areas that were recolonized after the LGM, diversity losses resulting from founder effects during rapid expansion have been suggested [1,21]. High genetic diversity within and strong differentiation between populations in southern areas (compared to that in the northern areas) are suggestive of the northward expansion of species [9,17,18,22–26]. Nonetheless, high genetic diversity can also be characteristic for contact zones where different lineages from distinct genetic sources mix [27,28]. Therefore, landscape history has to be taken into account to interpret the genetic pattern of plant populations successfully. Stable populations with long histories on-site (e.g., in glacial refugia) further indicate an accumulation of rare haplotypes, which are good indicators of refugial areas [29]. Because of stochastic processes, the identification of rare haplotypes is less likely in recently colonized habitats [30].

Regarding migrations during interglacial periods and after the LGM, mankind was an influential factor for most plant species in central Europe [31,32]. Natural postglacial reforestation was restrained by land use (e.g., grazing and agriculture), and suitable habitats for thermophilic plants were formed, including dry grasslands that evolved on sites with grazing livestock. Several plants further dispersed via migrations and human settlement after the LGM, during which humans took plants or seeds with them—intentionally or unintentionally with pastoralism [33–35]. In recent studies, the postglacial migration routes of anthropochorous species (plants and animals) have been linked to human migrations (e.g., migrations with livestock) [31,36–38].

To date, the postglacial histories of several tree species trees [9,11,14–18] in central Europe have been investigated, including those of arctic-alpine or rare species [39–50]. Whereas, studies about the postglacial histories of herbaceous lowland plants are scarce [13,21,26,51–53]. Common dry grassland species in Europe have hardly been analyzed despite the fact that the habitat is extremely species-rich and important to plant and animal conservation.

The succulent *Sedum album* L. (Crassulaceae) is a typical inhabitant of dry grasslands in alkaline and calcareous soils in central Europe. It can be found on rocks and shallow soils within the Festuco-Brometalia vegetation type and in pioneer vegetation on rocks within the Sedo-Scleranthetalia-group (Sedo albi-Veronicion dillenii) [54–56]. *S*. *album* can also be found in crevices on stony riverbanks and in forests with oak or olea in the Mediterranean. Further, the species immigrates to man-made habitats, including the stone walls of buildings and gardens in cities and villages [33,57–59]. Nowadays, *S*. *album* is common, and it is distributed over wide regions in temperate Europe and the Mediterranean.

In the present study, we investigated 34 populations of *S*. *album* in Europe using AFLP markers, chloroplast sequences (trnL-trnF), and chloroplast microsatellites to answer the following questions: 1) Can we infer the postglacial history of a common plant like *S*. *album* using standard methods (AFLPs and chloroplast markers)? 2) Was *S*. *album* introduced to central Europe after the LGM from glacial refugia in the southwest or southeast? 3) Can we detect cryptic refugia in central Europe? 4) What are the characteristics of the migration routes of this species to dry grasslands in central Europe?

## Materials and methods

### Species and collection

*Sedum album* L. (Crassulaceae) is a perennial herb, with succulent woody stem and succulent, linear-cylindrical to ovoid leaves. Seeds are long-term persistent and dispersed by rain and water. Shoots and leaves are very resistant to drought and can develop adventitious roots when they encounter favorable conditions or habitats. The flowers have white to pink petals and are either pollinated by insects, or self-pollinated.

*S*. *album* was used as ornamental, medicinal, and edible plant, and was proven to grow near human settlements [33,59–64]. The natural distribution range is all over Europe except for northern and eastern parts [55,65]. The eastern border of its distribution in Poland, Slovakia, Hungary, Romania, Bulgaria, and Turkey have been described [54,55]. In the south, this species can be found until northern Morocco, Algeria, and Tunisia. In the north the natural distribution is described in southern Sweden and Norway, whereas it was introduced in most parts of the UK and Ireland, and in parts of Sweden [55].

Chromosome numbers of 2n = 2x = 32, 34 and 38, as well as tetraploid individuals with 2n = 4x = 64, 68 have been described [66–68], which are not congruent with the segregates (*S*. *athoum*, *S*. *micranthum*) that have been described based on leaves (size and shape), flowers (size of flowers and shape of petals), distribution, and habitat conditions.

For the present study, 34 populations of *S*. *album* were sampled in dry grassland (Festuco-Brometalia) and rock slopes (Sedo albii-Veronicion dilenii) all over Europe (Table 1, S1 Table) except for the northern and eastern parts, where the populations might have been introduced [65]. *S*. *album* is not protected and we did not collect samples from protected areas, therefore no specific permissions were required. We avoided populations with characteristics of segregates, as well as populations near gardens and roof greenings to exclude the direct influence of horticultural S. *album* breeds. Fresh and young branches of six (GB) to 14 (S12) plants per population were sampled (Table 1) and stored in filter bags with silica gel until the material was frozen at -18°C. Voucher specimens are deposited at the Institute of Plant Sciences, University of Regensburg.

**Table 1:** 34 investigated populations of *S*. *album* in Europe. Given are identification (*ID*), country, number of individuals for AFLP (*nr*), microsatellite haplotypes (*ccmp*), trnL-trnF sequence haplotype (*seq*), groups revealed by AFLPs (*AFLP*): Central Europe East (CE-E) and West (CE-W), Western Europe (WE), Iberian Peninsula (IP), Southern Italy and western Alps (S-It & Al), Liguria (Lig); further, percentage of polymorphic fragments (*%PL*), diversity as Shannon Index (*SI*) and SSWP/n-1 (*SSWP*), down-weighted rare fragments (*DW*), private fragments (*priv*) and fragments restricted to two populations (*with*) are shown.

*ID*	*country*	*nr*	*ccmp*	*seq*	*AFLP*	*%PL*	*SI*	*SSWP*	*DW*	*priv*	*with*
A	Austria	11	a	H1	CE-E	20.22	0.10	12.92	9.85	0	SK
AND	Andorra	11	a	H12	IP	30.75	0.16	21.06	11.59	0	
BE	Belgium	11	a	H8	WE	22.71	0.12	15.08	7.61	0	
CZ	Czech Republic	11	a	H1	CE-E	26.87	0.15	4.75	10.09	0	
D1	Germany	11	a	H6	CE-W	9.70	0.04	7.36	9.87	2	
D2	Germany	12	a	H1	CE-W	10.35	0.06	6.27	7.25	0	
D3	Germany	12	f	H17	CE-W	13.3	0.05	15.78	12.31	1	SRB2
D4	Germany	12	a	H1	CE-W	17.73	0.07	8.39	7.70	0	
D5	Germany	11	a	H5	CE-W	19.94	0.09	12.06	9.17	0	
D6	Germany	11	a	H1	CE-E	19.39	0.10	13.66	8.06	0	
E1	Spain	10	c	H13	IP	22.71	0.12	12.99	9.45	0	
E2	Spain	12	a	H12	IP	33.24	0.17	22.49	11.88	0	
E3	Spain	13	a	H12	IP	33.24	0.17	21.17	14.22	2	E4
E4	Spain	12	a	H14	IP	26.87	0.14	17.62	12.07	0	E3
F1	France	12	a	H10	WE	26.32	0.12	15.76	10.32	0	F3
F2	France	11	a	H11	WE	23.82	0.12	15.75	7.40	0	
F3	France	11	a	H9	WE	27.7	0.14	18.96	10.53	1	F1, F4
F4	France	12	a	H12	WE	13.3	0.06	7.64	7.83	1	F3
F5	France	12	a	*	IP	21.88	0.10	6.00	7.62	0	
GB	Great Britain	6	a	*	WE	15.79	0.09	13.37		0	
I1	Italy	9	a	H3	S-It & Al	13.57	0.07	6.46	9.70	0	I8
I2	Italy	11	a	H4	CE-E	13.57	0.05	13.63	6.87	0	
I3	Italy	12	a	H1	CE-E	21.33	0.11	15.20	9.31	0	
I4	Italy	10	a	H1	CE-E	21.88	0.11	15.24	10.97	0	
I5	Italy	12	d	H16	Lig	13.3	0.05	6.56	14.74	3	2*I6
I6	Italy	11	e	H12	Lig	22.71	0.11	14.60	17.19	4	2*I5, I8
I7	Italy	14	a	H7	S-It & Al	23.55	0.12	14.68	10.04	1	
I8	Italy	12	a	H1	S-It & Al	29.64	0.15	20.28	13.82	2	I1, I6
PO	Portugal	11	b	H15	IP	9.42	0.04	5.00	15.68	6	
S1	Switzerland	12	a	H7	CE-W	22.71	0.12	12.54	8.97	0	
S2	Switzerland	12	a	H2	S-It & Al	15.79	0.06	7.33	10.52	1	
SK	Slovakia	12	a	H1	CE-E	21.05	0.10	19.71	10.02	0	A
SRB1	Serbia	11	f	H18	CE-E	22.99	0.11	14.87	12.70	0	SRB2
SRB2	Serbia	12	f	H18	CE-E	20.22	0.09	10.69	11.24	0	SRB1, D3

* no sequence data available

### DNA extraction

Approximately 5–8 leaves of each plant were homogenized with liquid nitrogen and DNA was extracted using the CTAB method [69] with minor adaptations [44]. DNA concentrations of all extractions were measured as transmission of optical density. A standard DNA concentration (7.8 ng/μl) was diluted, which was taken for all further investigations.

### AFLPs

A screening of 3 ×12 fluorescence-labeled primer combinations was carried out with eight individuals of four populations. Three high-resolving primer combinations were selected (D2: MseI-CAA/EcoRI-AAC, D3: MseI-CAT/EcoRI-AAG, D4: MseI-CAA/EcoRI-ACT).

AFLP analysis was conducted with 388 individuals in total following the protocol of Vos et al. [70] with minor variations [46]. About 50 ng of DNA was digested with EcoRI and MseI restriction enzymes (Fermentas) and ligated to adaptors (MWG) with T4 DNA Ligase (Fermentas) at 37°C for 2 h with a final step of 70°C for 15 min. Restriction-ligation and all following polymerase chain reactions were carried out in automated Thermocycler (Eppendorf) with Taq Polymerase (PeqLab). The first amplification step was conducted with a pair of one-base primers (MseI-C/EcoRI-A, MWG) in 30 cycles. Fluorescence labeled Mse primers were used for the final amplification with three-base primers (MseI-C/EcoRI-A, MseI-C/EcoRI-A, MseI-C/EcoRI-A, MWG); 25 cycles were run of which 10 cycles had a 1°C touchdown profile. The PCR products were precipitated with NaAc, EDTA, glycogen, and cold EtOH (96%, -20°C) and cleaned with cold EtOH (70%, -20°C). After the DNA pellet was dried, it was dissolved in Sample Loading Solution (Beckman Coulter) and charged with CEQ Size Standard 400 (Beckman Coulter). The capillary gel electrophoresis was run on an automated sequencer (CEQ 8000, Beckman Coulter) and the raw data were automatically analyzed with CEQ 8000 software (Beckman Coulter) for aligning the fragments according to CEQ Size Standard 400.

Clear and well-defined fragments were scored manually in the program BioNumerics v. 3.0 (Applied Maths). The fragments with sizes of 60–420 bp were processed and scored across all individuals as either present or absent. Both individuals and fragment sizes that did not give clear, defined, and reproducible fragments were excluded from the analysis. The resulting binary matrix was used for further statistical analysis.

To analyze the quality and reproducibility of the fragments, 55 individuals were replicated, which gave an error rate of 1.36% [71].

Genetic diversity within populations was calculated based on all markers that produced polymorphic fragments, percentage of polymorphic fragments, and Shannon Index (SI = ∑ pi ln pi) for each population [72], using the program POPGENE version 1.32 [73]. Sum of squares within populations (SSWP) was derived by analysis of molecular variance (AMOVA) in GenAlEx version 5 [74]. SSWP was divided by n-1 for each population to describe within population diversity independent from the number of individuals [75].

Rarity of fragments can indicate the level of isolation and differentiation [76]. Therefore, we tested populations for private fragments or shared fragments between two populations. We detected rarity as frequency-down-weighted markers (DW) for each population [30] with AFLPdat in R [77]. We randomly chose nine individuals per population in five iterations. Owing to few individuals, the GB population was excluded from this analysis.

Among population distances were estimated with the program AFLP-SURV [78] based on Nei’s standard (Ds) distance with non-uniform prior distribution of allele frequencies. Based on Ds distances, we constructed consensus neighbor-net graphs with Splits Tree [79].

The differentiation within populations, among populations, and among main groups revealed by neighbor-net graphs and Bayesian clustering was calculated in GenAlEx version 5 [74]. Based on pairwise genetic distances between individuals, population differentiation was inferred as PhiPT, which is an analogue of Wright’s F-statistics [80]. Further a hierarchical AMOVA was used to estimate differentiation between populations, within groups, and between groups [81].

Based on pairwise PhiPT values from AMOVA and pairwise geographic population distances (km) we conducted a Mantel-Test [82] in GenAlEx version 5 [74]. Correlations of genetic and geographic distance were tested using 999 permutations.

Genetically homogenous groups were inferred with Bayesian clustering in the program STRUCTURE version 2.2 for all individuals (K = 1–35) and two subgroups in separate runs (east: K = 1–21; west: K = 1–15) [83]. Allele frequencies were assumed to be correlated in an admixture model [84]. The number of groups was estimated using 10^5^ iterations, with a burn-in-period of 10^4^. For each predefined number of K, 10 iterations were run. From the resulting values of L(K) standard deviations and ΔK were calculated to affirm the most likely number of groups with STRUCTURE harvester [84,85]. The software CLUMPP was used to calculate similarity coefficients (Hʹ) between runs for the same K [86], and the output was used to visualize assignments of individuals to groups using the software DISTRUCT [87].

### cpDNA sequence analysis

The plastid trnL intron and trnL-F spacer sequences were chosen for a preliminary test using primers Tab c and Tab f [88], because they were the most variable regions of the chloroplast genome. TrnS-G intron [89] and rpl32-trnL(UAG) [90] were tested, but excluded for further analysis owing to low variability or difficult amplification.

Polymerase chain reactions (PCRs) were carried out in 10 μl reaction volumes containing: 3 μl DNA (7.8 ng/μl), 0.4 μl of each primer (10 mM), 1 μl BSA, 1 μl dNTPs (5 mM), 1 μl PCR Buffer (10X), 1 μl MgCl_2_ (50 mM), 0.08 μl Taq (PeqLab) and 2.12 μl water for molecular biology (Roth). The reactions were performed in a thermal cycler (Eppendorf) with 35 cycles of denaturing at 95°C (30 s), annealing at 52°C (90 s) and extension at 72°C (4 min), followed by a final extension step at 72°C (7 min), and hold at 4°C.

The PCR products were purified using 4 μl of a mixture of Exonuclease (0.08 μl, 20 U/μl), SAP (0.8 μl, 1 U/μl) and water (3.12 μl). Purification was conducted at 37°C (30 min) and concluded with a step at 95°C (5 min).

Cycle sequencing was performed with 3 μl purified product, 0.6 μl Buffer (20X, Beckman Coulter), 0.6 μl primer (10 μM), 1.4 μl DTCS Master Mix (Beckman Coulter) and 4.4 μl water for molecular biology (Roth). The PCR products were precipitated with Glycogen (20 mg/ml), NaAc (3M, pH 5.2), Na_2_EDTA (100 mM, pH 8) ice-cold EtOH (96%) and washed with EtOH (70%). These products were subjected to capillary gel electrophoresis on an automated sequencer (CEQ 8000, Beckman Coulter).

Two individuals per population were used for this analysis. All the samples were amplified in both directions (with Tab f and Tab c), but an overlap could not be achieved in any of the samples. To reassure the stability of the sequences, individuals were sequenced twice and consensus sequences were used for further analyses. Populations F5 from France and GB from Great Britain were excluded from further analyses because we failed to produce two appropriate sequences per population.

We compared the resulting sequences with those in GenBank using Blast [91]. Alignment was conducted using Clustal Omega [92] and adjusted manually with BioEdit Sequence Alignment Editor 7.2.0 [93]. The sequences were submitted to Genbank (S2 Table).

Concatenated cpDNA sequences were used to build a network under statistical parsimony (as implemented in Clement et al. [94]) in TCS v1.21 with gaps coded as missing. The connection limit was set at 120 steps to include the out-group *Sedum dasyphyllum* from Barcelona (BCN) [95].

Maximum parsimony (MP) heuristic searches were conducted in PAUP* 4.0a [96] with TBR branch swapping in action and gaps coded as missing. Confidence was assessed by bootstrap analysis with 1000 bootstrap replicates and 100 random addition sequence replicates per bootstrap (with a limit of 10 sec per sequence replicate).

### Chloroplast microsatellites

Universal conserved chloroplast microsatellite primers (ccmp1, ccmp2, ccmp3, ccmp5, ccmp6, ccmp7, ccmp8, ccmp9, and ccmp10) were tested in an initial screening of four individuals [97]. Ccmp2, ccmp3, ccmp6, ccmp7, ccmp8, and ccmp10 yielded consistent products. Among these primers, only ccmp2, ccmp3, and ccmp6 were variable and used for further investigations using two individuals per population. Universal M13-tailed primers were labeled with fluorescent dyes DY-571, Cy 5.5, and Cy 5 (MWG). Amplifications were conducted in 10-μl reaction volumes containing 2 μl DNA (7.8 ng/μl), 0.1 μl ccmp-forward primer (1 mM), 0.15 μl universal fluorescence labeled ccmp-forward primer (10 mM), 0.15 μl ccmp-reverse primer (10 mM), 0.4 μl dNTPs (5 mM), 1 μl PCR Buffer (10X), 0.05 μl Taq (PeqLab) and 6.25 μl water for molecular biology (Roth). The reactions were run in a thermal cycler (Eppendorf) with a denaturation step at 94°C (5 min), followed by 34 cycles of denaturing at 94°C (1 min), annealing at 50°C (1 min) and extension at 72°C (1 min), and a final extension step at 72°C (8 min), and hold at 4°C.

For each individual, 1 μl of the PCR products of each primer was mixed and added to Sample Loading Solution, charged with an internal standard (CEQ Size Standard 400) and run on an automated capillary sequencer (CEQ 8000, Beckman Coulter). Chloroplast haplotypes were defined by fragment lengths using CEQ 8000 software. A haplotype network was built under statistical parsimony in TCS v1.21 [94]. Geographic sampling regions were used to analyze group structure of sequence and microsatellite haplotypes.

## Results

### AFLP analysis

**Fig 1:**
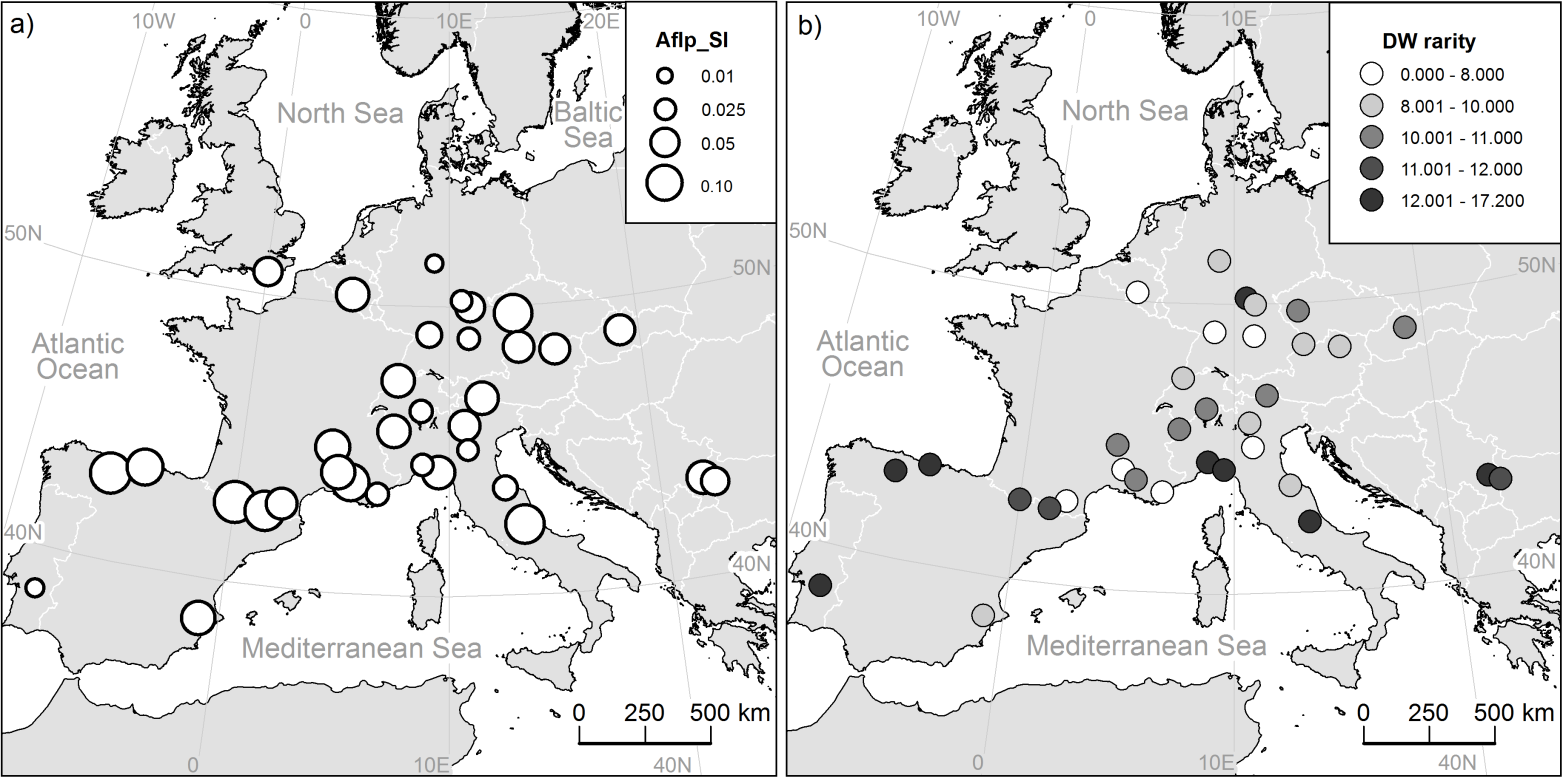
Maps of all investigated populations of *S*. *album*. a) Level of genetic diversity (AFLP_SI) is indicated by circle size b) levels of rarity (DW rarity) for each population are indicated by color.

AFLP analysis of 385 individuals out of 34 populations resulted in 361 fragments (D2: 135, D3: 116, D4: 110), of which 96.4% were polymorphic. Twenty-four fragments were private in single populations (Table 1). Genetic diversity ranged from SI = 0.1724 (E3) to SI = 0.391 (D1). Populations from the southern regions showed higher diversity than those from the northern and central Europe (Fig 1A). Likewise, the rarity of fragments was high in the southern populations (max. DW = 17.19 in I6 and max. private fragments = 6 in PO) and lower in the northern and the central populations (min. DW = 6.87 in I2) (Fig 1B). Private fragments were accumulated in populations D1 (Germany), I5 and I6 (Liguria), PO (Portugal), E4 (northern Spain), and I8 (Central Italy).

**Fig 2:**
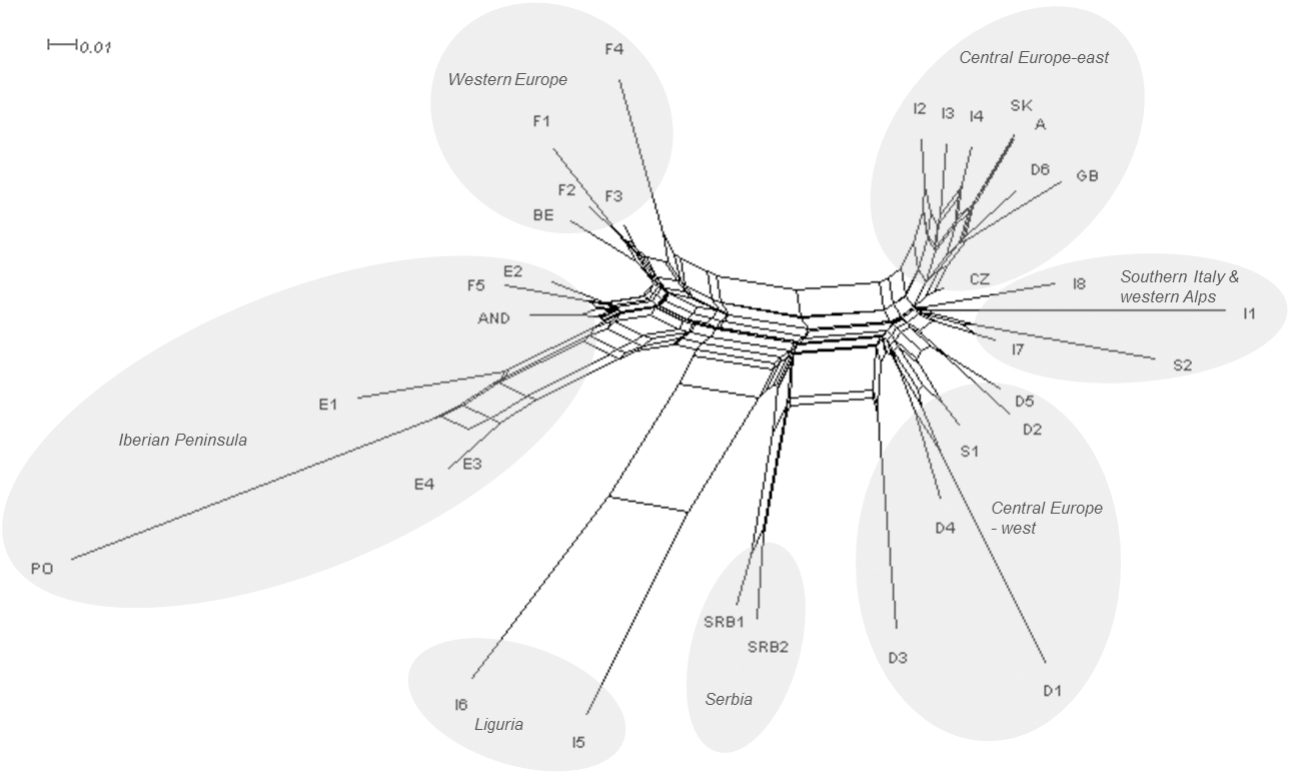
Neighbor-net graph based on 385 *S*. *album* individuals analyzed with AFLPs.

In the neighbor-net graph (Fig 2) the separation of southwestern Europe (Spain and Portugal), western Europe, Serbia, Liguria, and central Europe with Italy was evident. Central Europe and Italy formed one group and a further division in three subgroups was possible: central Europe-west consisted of populations from western Germany and Switzerland, Central Europe-east consisted of populations from Austria, Czech Republic, Germany, Italy, Slovakia, but also Great Britain. With the exception of the population from Great Britain, populations formed one lineage from northeast Italy northward to Germany and eastward to Czech Republic and Slovakia. Populations from the middle of the Apennine Peninsula and the western Alps (Italy and Switzerland) formed a third group. Populations I5 and I6 from northwestern Italy (Liguria) were segregated from the rest of the Italian group and clustered between the populations from Serbia and those from Iberia. D3 from Central Europe was separated in the network from its geographic neighbors and situated between the populations from Serbia and western central Europe.

**Fig 3:**
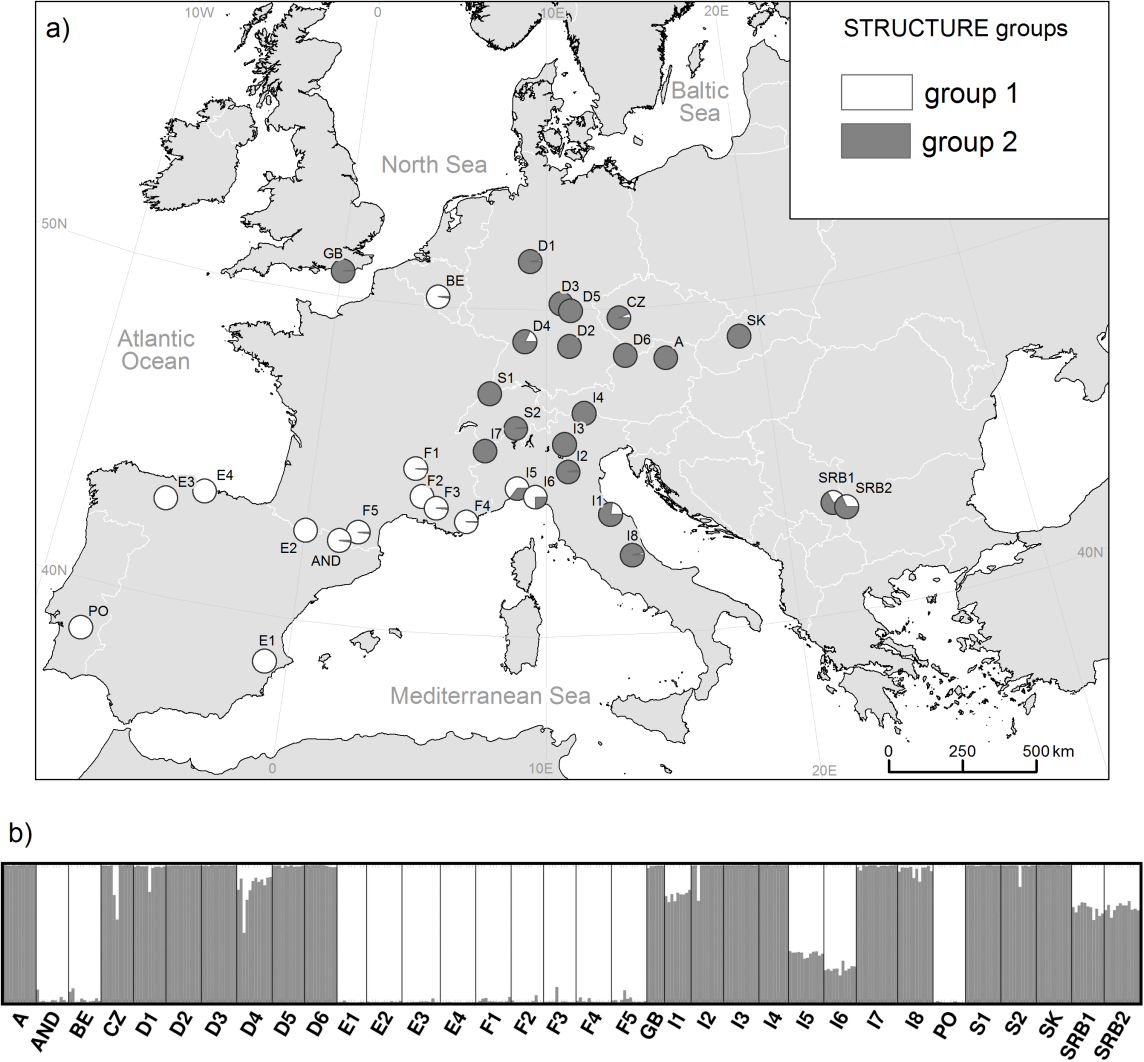
Inference of population structure of *S*. *album* obtained from Bayesian analysis in STRUCTURE. a) Geographic distribution of Bayesian groups (K = 2) and b) cluster assignment of each individual.

Differentiation was high between populations in general (72.3%, Table 2). We found 32.7% differentiation between groups, which have been determined by neighbor-net analysis and were mainly congruent with geographic regions. We omitted the regions Liguria and Serbia (incl. D3) from the AMOVA and population structure analysis, because those regions contain a large number of STRUCTURE groups (Fig 3) and the assignment to only one group is, therefore, not accurate. The AMOVA of STRUCTURE groups revealed similarly high differentiation between both groups (29.8%).

**Table 2:** Analysis of molecular variance (AMOVA). AMOVA was conducted for main geographic regions (neighbour-net regions) and STRUCTURE groups. Differentiation of the regions Iberian Peninsula, Serbia, Western and Central Europe were tested in different combinations; populations from Liguria were excluded from the analysis.

	d.f.	sum of squares	est. var	total variance (%)
**Overall analysis**				
among populations	34	13379.442	34.337	72.3
among individuals within populations	353	4653.576	13.183	27.7
**Analysis of neighbour-net regions**				
among regions	6	6700.3	16.29	32.7
among populations within regions	27	6555.33	20.36	40.8
among individuals within populations	351	4641.58	13.22	26.5
**Analysis of STRUCTURE groups (without Liguria and Serbia)**				
among regions	1	2922.16	16.07	29.8
among populations within regions	28	8066.74	24.33	45.2
among individuals within populations	309	4157.1	13.45	25.0
**Iberia <-> Western E & Central E & Serbia**				
among regions	1	1895.484	11.976	21.6
among populations within regions	32	11360.145	30.218	54.5
among individuals within populations	351	4641.576	13.224	23.9
**Iberia & Western E <-> Central E & Serbia**				
among regions	1	2734.036	13.553	24.8
among populations within regions	32	10521.593	27.894	51.0
among individuals within populations	351	4641.576	13.224	24.2
**Serbia (without D3) <-> Central E & Iberia**				
among regions	1	846.14	10.583	18.7
among populations within regions	31	11841.109	32.638	57.6
among individuals within populations	340	4572.66	13.449	23.7

To reveal the influence of other regions on central European populations, we tested differentiations of all combinations of the regions against each other (Table 2). The groups “Western Europe” and “Iberian Peninsula” versus “Central Europe” with “Serbia” and “Southern Italy & western Alps” showed the highest differentiation. Even without the population D3, which was genetically similar to Serbia in all the analyses, a close connection of populations from central to eastern parts of central Europe and southern Europe was found. Notably, the differentiation between populations was extraordinary high in the groups from the Iberian Peninsula (61.0%) and between populations from Liguria (75.0%; data analysis of single neighbor-net regions not shown).

We found significant correlation of genetic and geographic distances with Mantel-Test over the whole dataset (r = 0.362, p = 0.001) indicating isolation by distance among populations. Isolation by distance was further present in two of the main network groups (Central Europe, Iberian Peninsula), but absent between the populations from Western Europe (data not shown).

**Fig 4:**
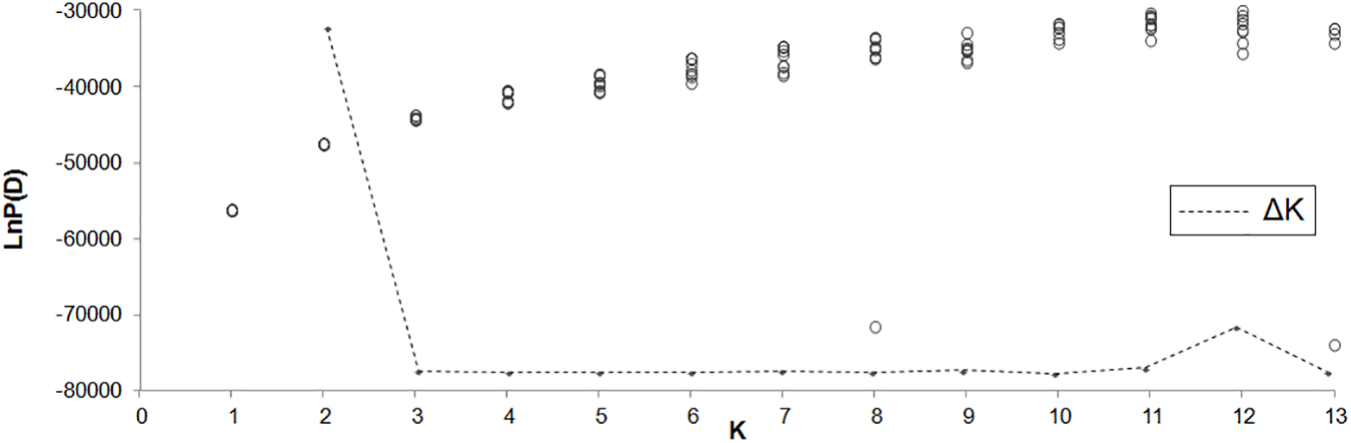
Bayesian clustering of AFLP data with STRUCTURE. Most probable subdivision was into K = 2.

Bayesian analysis distinguished the most probable subdivision in two clusters (ΔK = 412.2 and Hʹ = 0.993; Fig 4). A western lineage was dominant in populations from southern, western, and northern Europe, including Spain, Portugal, Andorra, France, and Belgium (Fig 3A and 3B). An eastern lineage was found in all Central European populations (Germany, Austria, Switzerland, UK, northern Italy and east of the Apennine mountain range, Czech Republic, and Serbia). High content of both lineages could be detected in populations I5 and I6 from Liguria, I1 from central Italy, D4 from western Germany and both populations from Serbia.

When both lineages were analyzed separately in Bayesian analyses, the highest probability in the western lineage of was detected for K = 3 (ΔK = 47.0 and Hʹ = 0.995, S1A Fig) and K = 7 (ΔK = 44.0 and Hʹ = 0.347, S1B Fig); individuals of the eastern lineage are most probably assigned to K = 3 (ΔK = 102.2 and Hʹ = 0.975, S2 Fig).

### Chloroplast sequences

Chloroplast sequences of regions trnL-trnF in 32 individuals of *S*. *album* resulted in concatenated sequences of 750 bases (gaps and missing data excluded). Forward strand (revealed by Tab c) was 324 bases long and had 14 mutation sites (7 single-nt polymorphisms, two single-nucleotide (nt) indels, two 2-nt indels, two 3-nt indels and one 8-nt indel). Reverse strand (revealed by Tab f) resulted in 426 bases with 16 variable positions (12 single-nt polymorphisms, one single-nt indel, two 2-nt indels, and one highly variable 12-nt indel). Overall, we detected 19 single-nt polymorphisms and 11 indels resulting in 18 sequence haplotypes (S2 Table).

**Fig 5:**
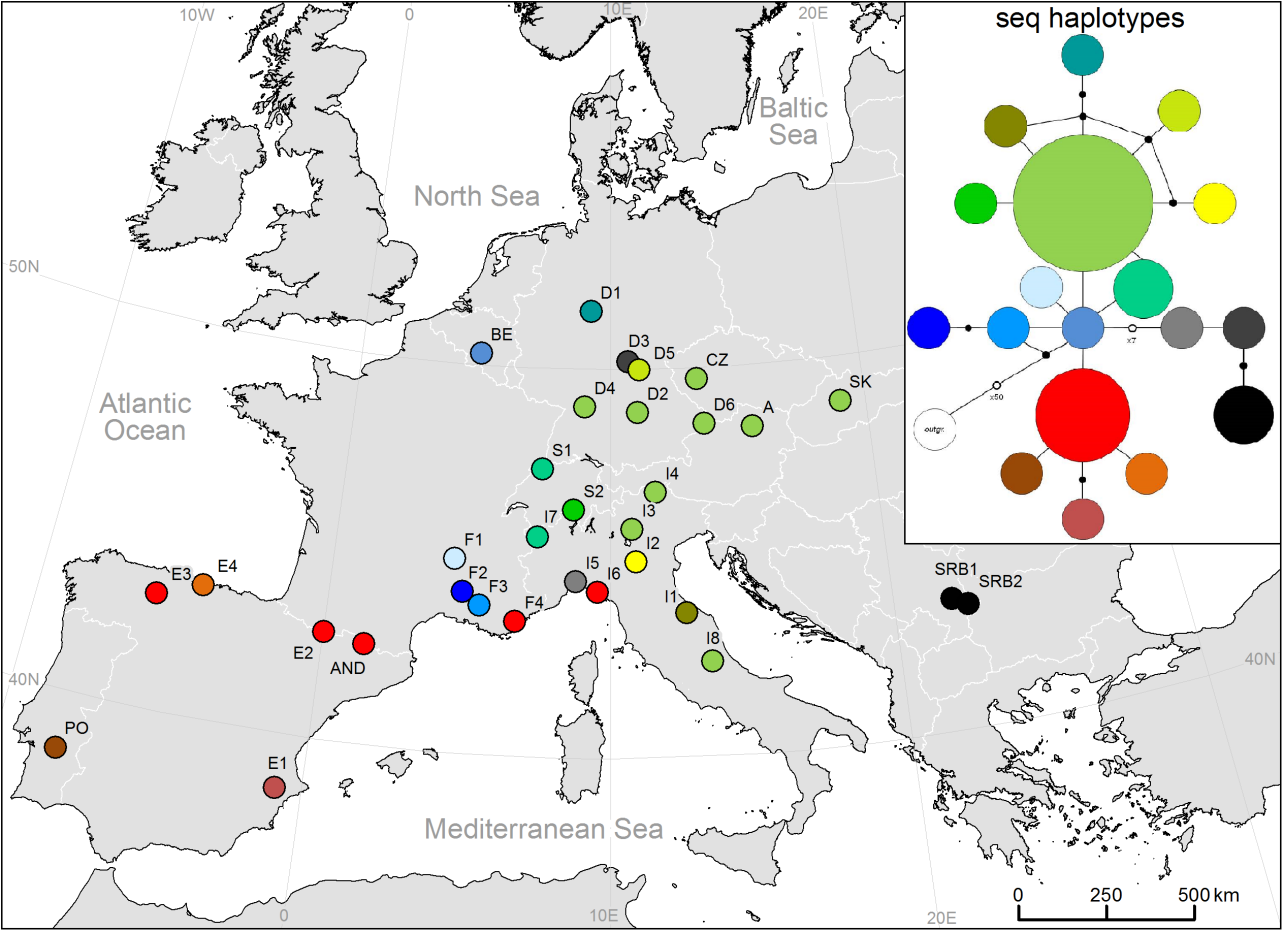
cpDNA haplotypes revealed by sequence analysis of trnL-trnF chloroplast region. Relationships between haplotypes are shown by colors in the statistical parsimony network; filled nodes explain two steps difference; unfilled nodes explain differences according to nearby numbers.

**Fig 6:**
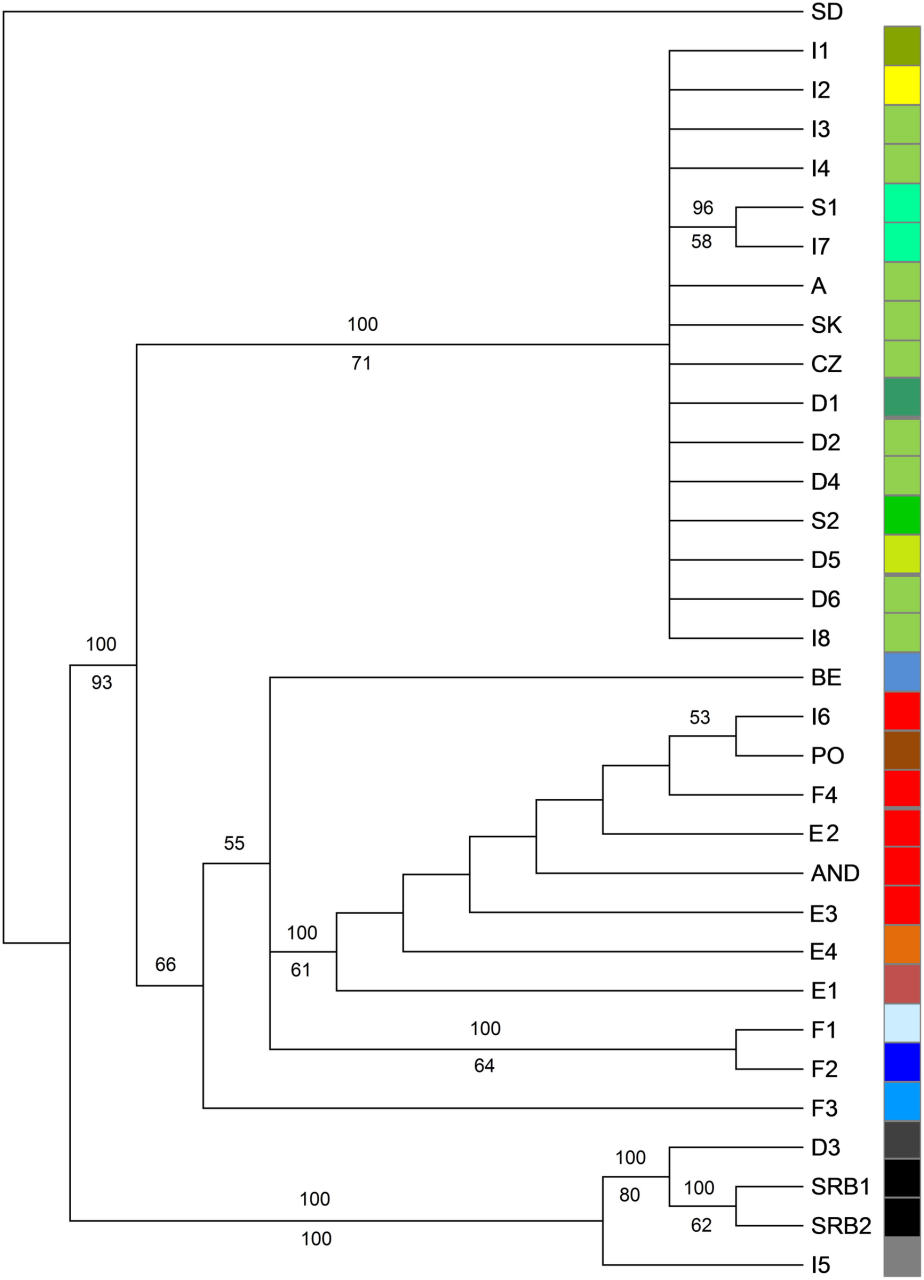
Majority rule consensus tree of 29947 equally parsimonious trees based on cpDNA sequences (750 bases). Number above branches refer to frequency of occurrences in the 50% majority rule consensus tree, and number below branches refers to the bootstrap support values from maximum parsimony analysis >50%. The tree was manually rooted on the branch separating *S*. *dasyphyllum*.

A group of nine populations from north-east Italy (I3, I4, I8), Austria (A1), Czech Republic (CZ), Slovakia (SK) and Germany (D2, D4, D6) shared haplotype H1 (Fig 5; Table 1). Furthermore, single populations from north-western Italy (I6), southern France (F4) and the Iberian Peninsula (AND, E2, E3) were represented by haplotype H12. A clear separation of populations from Serbia (SRB1, SRB2), Germany (D3) and Ligura (I5) was detected in the TCS network (Fig 5), which was congruent with the pattern of the maximum parsimony (MP) tree and (Fig 6) the neighbor-net graph (Fig 2).

Maximum parsimony analysis yielded 29947 equally parsimonious trees. For a 50% majority rule gene tree *S*. *dayphyllum* was manually set as an outgroup [95] (Fig 6). The trees reflected the geographic locations of the populations. The clade with Serbian populations, including populations D3 and I5, were highly distinct. Further, highly supported clades distinguished populations on the Iberian Peninsula and Western Europe from those in central Europe and Italy.

### Chloroplast microsatellites

**Fig 7:**
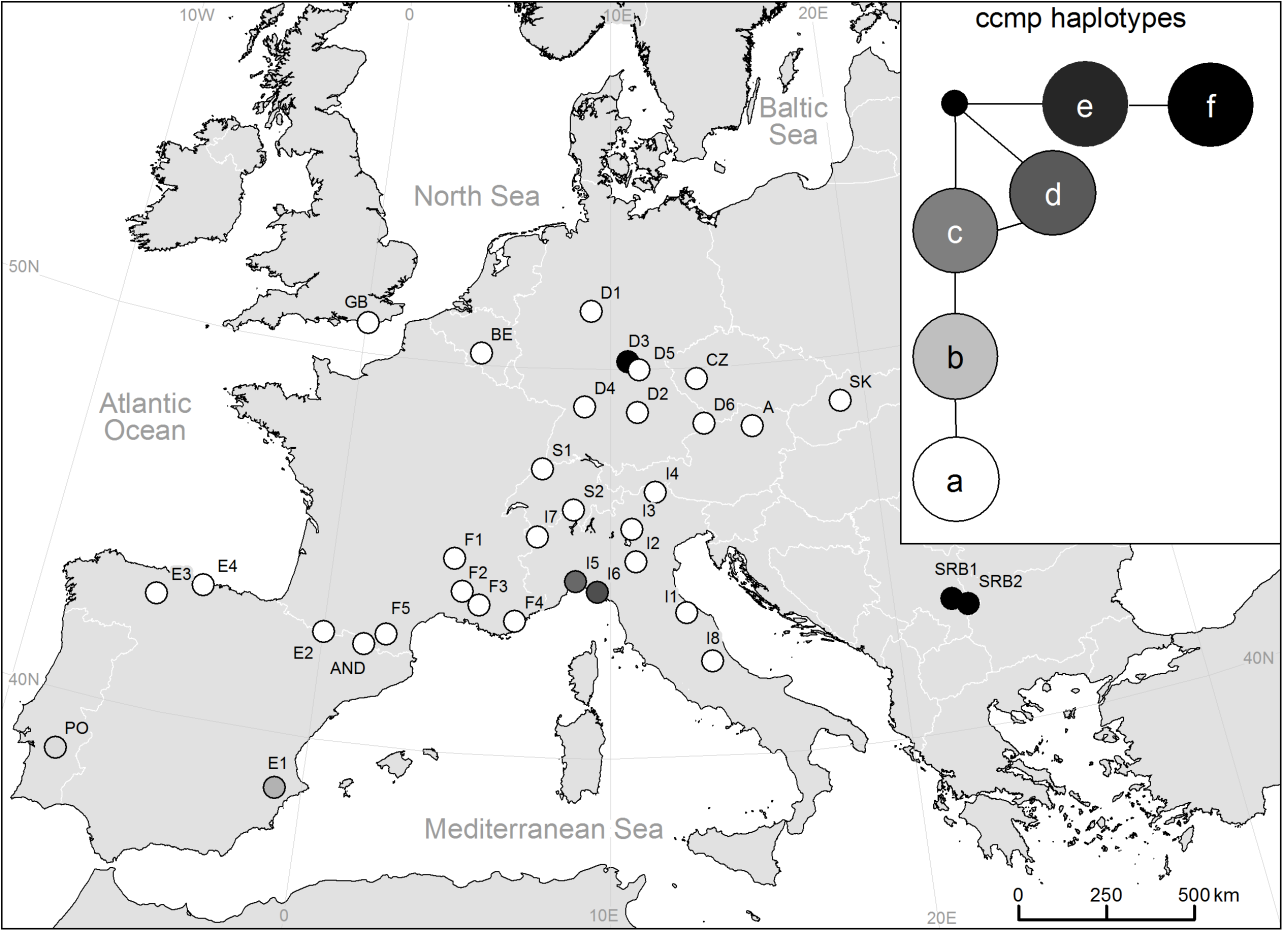
cpDNA haplotypes revealed by ccmp analysis. Relationship between haplotypes is shown in the network.

Chloroplast microsatellites revealed six haplotypes (Fig 7, Table 1), whereas we could detect four polymorphisms at locus ccmp1, three at ccmp2 and two at ccmp3. Haplotype a was present in the main group of 28 populations. Haplotype f was observed in both the Serbian populations (SRB1 and SRB2) and in D5. Four haplotypes represented single populations (E1, I5, I6, and PO). Relations of the haplotypes are shown in a network (Fig 7).

## Discussion

We found high resolution and clear geographic structure in *S*. *album* populations in Europe with AFLPs, chloroplast sequence analysis and chloroplast microsatellites. A clear east/west subdivision in the investigated area, with a south-northward contact zone in western Europe was detected. AFLPs supported the results of chloroplast microsatellites and sequences (single exceptions discussed below), consistent with the findings of other studies for plants and animals [37,98].

The Iberian Peninsula populations of *S*. *album* showed high differentiation and high rarity levels. Both values suggest that populations are more isolated than those from the other regions. The microsatellites revealed distinct haplotypes of populations E4 from northern Spain and PO from Portugal. The divergences of clades from the Iberian Peninsula and western Europe seem to be ancient compared to the clades from central Europe and Italy. The Iberian Peninsula has been suggested as one of the main glacial refugia for *Alnus glutinosa* [19], *Helianthemum* sp. [13] and *Rosa pendulina* [7]. In all these studies, ancient haplotypes have been found on the Iberian Peninsula.

Populations AND, E2, and F5 are situated in the Pyrenees. They were clearly assigned to the Iberian Peninsula group and belonged to a distinct cluster in the Bayesian analysis of the western group (K = 7, S1B Fig). Moreover, AND and E2 had extraordinary high genetic diversity and rarity. Closed mountain valleys can harbor glacial refugia [3,27,99], and the findings of the present study support this, based on the identification of glacial refugia for *S*. *album* in the Pyrenees.

Populations from western Europe (BE, F1-F4) are closely related to populations from the Iberian Peninsula (E1- E4, PO). This northward line in western Europe represents the genetic border with populations in central Europe. Based on high genetic diversity within populations, it is possible that *S*. *album* has survived the LGM in lowland central Europe refugia, as suggested for *Helianthemum nummularium* [13]. Population F3 is the ancestor of the western group, according to the results of the cpDNA sequence analysis. It further showed that high rarity and diversity, which indicate high isolation in a cryptic refugium. Cryptic refugia have been shown for many plants surviving the ice ages in scattered areas with more suitable microclimate [5,6]. This was demonstrated in recent studies for several shrub and herb species in Central Europe [7,100,101]. The high amplitude of ecological niches that *S*. *album* inhabits makes survival in northern cryptic refugia possible.

Although population BE situated in north-central Europe also had a basic position in the cpDNA analysis (Fig 5 and Fig 6), the rarity of fragments was low and diversity was high, which both is contradictory to an ancestral population. A recent single dispersal event from an ancient source population would explain the ancestral cpDNA haplotype. Frequent gene flow from nearby populations via pollination Frequent gene flow via pollination by nearby populations would further explain the low rarity and high diversity levels. However, we could not detect isolation by distance within the group in Western Europe. Differentiation between the populations in general was low and rarity was moderate. Therefore, we suggest that most populations in Western Europe are exposed to frequent gene flow and constitute a contact zone for lineages from Iberia and Central Europe-east, with greater influence from Iberia.

Central European and Italian populations share one microsatellite haplotype, except for I5 and I6 from Liguria. Additionally, nine populations from Central Europe and Italy shared one sequence haplotype; further haplotypes had a close relation (except for I5 and I6 from Liguria). This group forms one derived cluster in the MP tree. In the AFLP network, populations from Central Europe and Italy could be divided in three subgroups (Central Europe-east, Central Europe–west and Italy with western Alps). Owing to poor differentiation, and low rarity and diversity, a young colonization history can be assumed for most populations from Switzerland (S1, S2) and Germany (D1, D2, D4, D5, D6).

Population D3 shares one microsatellite type with Serbian populations and is closely related to the sequence haplotype of Serbian populations. It showed high rarity, but low genetic diversity, which would be explained by a single dispersal event inducing a bottleneck and high isolation.

High rarity was also found in populations from the Czech Republic (CZ) and Slovakia (SK), along with high diversity and the most ancient cpDNA haplotype (H1) that is distributed widely in Central Europe. All analyses point towards cryptic refugia for *S*. *album* in the Czech Republic and Slovakia.

Italian populations, which are situated north and east of the Apennine (I2, I3, and I4), showed close genetic relations northward to Germany and eastward to the Czech Republic and Slovakia (S2 Fig). Therefore, gene flow or migration of *S*. *album* along the river valley of the Danube can be assumed.

Populations situated near the Alps (I7 and S2, from Italy and Switzerland, respectively) have a strong relation to populations from the middle of the Apennine peninsula. We suggest that at least these parts of the Alps are colonized by lowland southern Italian source populations after the LGM [47]. Furthermore, alpine populations have a strong connection to Central European populations and, therefore, share a colonization history with populations from the northern parts of Europe.

Mountain ranges act as contact zones for different animal and plant species [102–105]. In particular, the region Liguria is known to be a potential contact zone for lineages from different source populations of *Anthyllis montana* [42,43]. Notwithstanding, populations I5 and I6 showed extraordinary high rarity and differentiation, which both indicates strong isolation, contradictory to the theory of a contact zone. Survival in small-scaled bedrocks near the Alps was already assumed for different alpine species [13,30,106]. Supposedly, Liguria also harbored glacial refugia in the mountain ranges or nearby valleys for *S*. *album*.

Population I5 from north-eastern Italy (Liguria) had a strong connection to populations from Serbia. In AFLP analysis, it was also related to the group on the Iberian Peninsula. In *Anthyllis montana* [42], a strong connection of populations from Liguria with eastern populations—from Greece and the Balkans—has been previously shown. This connection was also present in red deer [107]. A similar picture can be drawn for *S*. *album* in the nearby population I6 in Liguria. It has a strong connection to the south-western group in Spain/Portugal, but also has some similarities with the Serbian populations. A strong relation between populations along the Mediterranean coast, from Spain and France to the middle of the Apennine Peninsula, and further eastward to Serbia was detected; this potentially reflects the northern distribution limit of *S*. *album* that first expanded west- and eastward after the LGM, before the ice and steppe regions moved further north. Moreover, dispersal along with human migrations are important considerations due to the ethnobotanical use of *S*. *album* and the longevity of seeds and shoots. Human migrations, in particular with La Hoguette culture, were documented from south-eastern Europe along the middle of the Apennine Peninsula and the Mediterranean coast [31,100], resembling the migration route of *S*. *album*.

Four populations (SRB1, SRB2, D3, and I5) constitute the most ancestral group in the MP tree. High rarity but low genetic diversity in population D3 indicates a singular long-distance dispersal event from a source in south-eastern Europe. Both Serbian populations (SRB1, SRB2) show low rarity and differentiation among populations, as revealed by the AFLP analysis. In the AFLP analysis, Central European populations were more related to Serbian populations than to populations from Iberia and Western Europe. It is possible that Serbia was colonized from source populations farther south or east on the Balkan peninsula (e.g. Greece or Bulgaria) after the LGM, and populations migrated to Central Europe. The influence of the eastern lineage on Central Europe is observed in the strong relation to population I5 and the close relation to central and northern populations, as indicated in the AFLP analysis.

Besides the clear east/west division, there was an admixture of the main AFLP lineages in populations from Germany (D4), Liguria (I5, I6), Italy (I1), and Serbia (SRB1, SRB2). Multiple colonization events would explain the observed pattern in D4 and I1. For the postulated glacial refugia in Liguria and Serbia, the combination of long-term isolation and recent frequent gene flow by pollination would explain the pattern of AFLPs and cpDNA [7]. A clear subdivision of populations from Liguria and Serbia emerged from the further subdivision of the main lineages (S1 and S2 Figs), further emphasizing the strong isolation of this area.

## Conclusion

A combined analysis of the conserved chloroplast regions and AFLP markers with high resolution made it possible to infer the historical processes of *S*. *album* populations in Europe. *S*. *album* survived in glacial refugia on the Iberian and the Apennine Peninsula, and probably in eastern parts of Central Europe—as shown for several other species [3,9,108]. We found candidate areas for glacial survival in closed valleys in the Pyrenees and the mountains of Liguria, and cryptic refugia in France, the Czech Republic and Slovakia.

Recently two studies on dry grassland species demonstrated the colonization of Central Europe from source populations on the Iberian Peninsula after the LGM [100,101]. In contrast, we showed that *S*. *album* migrated to western and north-western parts of Europe from Spain/Portugal, but grasslands in Central Europe were primarily recolonized by cryptic source populations or source populations coming from the Apennine Peninsula and eastern Central or south-eastern Europe.

## Supporting information

S1 Fig Bayesian analysis of the western subgroup. Most probable subdivision of the western group was a) K = 3 and b) K = 7.
(TIF)

S2 Fig Bayesian analysis of the eastern subgroup. Most probable subdivision of the eastern group was K = 3.
(TIF)

S1 Table 0/1- Matrix from AFLP analysis. Raw data of 385 individuals in 34 populations that are used for all further analyses; included are geographic coordinates for each population.
(XLSX)

S2 Table Polymorphic sites of trnL-trnF chloroplast regions. Concatenated sequences of *S*. *album* individuals from 32 populations that were used for all further analyses of chloroplast sequences.
(DOCX)

## References

[1] Hewitt GM (2000) The genetic legacy of the Quaternary ice ages. Nature 405: 907–913. doi: 10.1038/35016000 10879524 10.1038/35016000

[2] Mithen S (2006) After the ice: a global human history, 20,000–5000 BC. London: Harvard University Press. 664 p.

[3] Taberlet P, Fumagalli L, Wust-Saucy A-G, Cosson J-F (1998) Comparative phylogeography and postglacial colonization routes in Europe. Molecular Ecology 7: 453–464. 9628000 10.1046/j.1365-294x.1998.00289.x

[4] Kajtoch Ł, Cieślak E, Varga Z, Paul W, Mazur MA, Sramkó G, et al (2016) Phylogeographic patterns of steppe species in Eastern Central Europe: a review and the implications for conservation. Biodiversity and Conservation 25: 2309–2339.

[5] Stewart JR, Lister AM (2001) Cryptic northern refugia and the origins of the modern biota. Trends in Ecology & Evolution 16: 608–613.

[6] Stewart JR, Lister AM, Barnes I, Dalén L (2010) Refugia revisited: individualistic responses of species in space and time. Proceedings of the Royal Society of London B: Biological Sciences 277: 661–671. 10.1098/rspb.2009.1272 PMC2842738 19864280

[7] Daneck H, Fér T, Marhold Fls K (2015) Glacial survival in northern refugia? Phylogeography of the temperate shrub *Rosa pendulina* L.(Rosaceae): AFLP vs. chloroplast DNA variation. Biological Journal of the Linnean Society 119: 704–718.

[8] Comes HP, Kadereit JW (1998) The effect of Quaternary climatic changes on plant distribution and evolution. Trends in Plant Science 3: 432–438.

[9] Dumolin-Lapègue S, Demesure B, Fineschi S, Le Come V, Petit RJ (1997) Phylogeographic structure of white oaks throughout the European continent. Genetics 146: 1475–1487. 9258689 10.1093/genetics/146.4.1475 PMC1208090

[10] Rendell S, Ennos RA (2003) Chloroplast DNA diversity of the dioecious European tree *Ilex aquifolium* L. (English holly). Molecular Ecology 12: 2681–2688. 12969471 10.1046/j.1365-294x.2003.01934.x

[11] Palmé AE, Vendramin GG (2002) Chloroplast DNA variation, postglacial recolonization and hybridization in hazel, *Corylus avellana*. Molecular Ecology 11: 1769–1779. 12207726 10.1046/j.1365-294x.2002.01581.x

[12] Cottrell JE, Krystufek V, Tabbener HE, Milner AD, Connolly T, Sing L, et al (2005) Postglacial migration of *Populus nigra* L.: lessons learnt from chloroplast DNA. Forest Ecology and Management 206: 71–90.

[13] Soubani E (2010) Systematics, phylogeography and multiple origins of morphs in two species complexes belonging to Cistaceae, Helianthemum oelandicum and H. nummularium. [Dissertation]. Lund: Lund University. 178 p.

[14] Konnert M, Bergmann F (1995) The geographical distribution of genetic variation of silver fir (*Abies alba*, Pinaceae) in relation to its migration history. Plant Systematics and Evolution 196: 19–30.

[15] Heuertz M, Fineschi S, Anzidei M, Pastorelli R, Salvini D, Paule L, et al (2004) Chloroplast DNA variation and postglacial recolonization of common ash (*Fraxinus excelsior* L.) in Europe. Molecular Ecology 13: 3437–3452. doi: 10.1111/j.1365-294X.2004.02333.x 15488002 10.1111/j.1365-294X.2004.02333.x

[16] Magri D (2008) Patterns of post‐glacial spread and the extent of glacial refugia of European beech (*Fagus sylvatica*). Journal of Biogeography 35: 450–463.

[17] Ferris C, King RA, Väinölä R, Hewitt GM (1998) Chloroplast DNA recognizes three refugial sources of European oaks and suggests independent eastern and western immigrations to Finland. Heredity 80: 584–593. 9650279 10.1046/j.1365-2540.1998.00342.x

[18] Grivet D, Petit RJ (2003) Chloroplast DNA phylogeography of the hornbeam in Europe: evidence for a bottleneck at the outset of postglacial colonization. Conservation Genetics 4: 47–56.

[19] King AR, Ferris C (1998) Chloroplast DNA phylogeography of *Alnus glutinosa* (L.) Gaertn. Molecular Ecology 7: 1151–1161.

[20] Avise JC (2009) Phylogeography: retrospect and prospect. Journal of Biogeography 36: 3–15.

[21] Grivet D, Petit RJ (2002) Phylogeography of the common ivy (*Hedera* sp.) in Europe: genetic differentiation through space and time. Molecular Ecology 11: 1351–1362. 12144657 10.1046/j.1365-294x.2002.01522.x

[22] Demesure B, Comps B, Petit RJ (1996) Chloroplast DNA phylogeography of the common beech (*Fagus sylvatica* L.) in Europe. Evolution 50: 2515–2520. doi: 10.1111/j.1558-5646.1996.tb03638.x 28565658 10.1111/j.1558-5646.1996.tb03638.x

[23] Després L, Loriot S, Gaudeul M (2002) Geographic pattern of genetic variation in the European globeflower *Trollius europaeus* L. (Ranunculaceae) inferred from amplified fragment length polymorphism markers. Molecular Ecology 11: 2337–2347. 12406244 10.1046/j.1365-294x.2002.01618.x

[24] Hedrèn M (1997) Genetic variation and hybridization in Swedish *Schoenus* (Cyperaceae). Plant Systematics and Evolution 204: 21–37.

[25] Hewitt GM (1999) Post-glacial re-colonization of European biota. Biological Journal of the Linnean Society 68: 87–112.

[26] Rendell S, Ennos RA (2002) Chloroplast DNA diversity in *Calluna vulgaris* (heather) populations in Europe. Molecular Ecology 11: 69–78. 11903905 10.1046/j.0962-1083.2001.01413.x

[27] Petit RJ, Aguinagalde I, Beaulieu J-Ld, Bittkau C, Brewer S, Cheddadi R, et al (2003) Glacial refugia: hotspots but not melting pots of genetic diversity. Science 300: 1563–1565. doi: 10.1126/science.1083264 12791991 10.1126/science.1083264

[28] Walter R, Epperson BK (2005) Geographic pattern of genetic diversity in *Pinus resinosa*: contact zone between descendants of glacial refugia. American Journal of Botany 92: 92–100. doi: 10.3732/ajb.92.1.92 21652388 10.3732/ajb.92.1.92

[29] Paun O, Schönswetter P, Winkler M, Consortium I, Tribsch A (2008) Historical divergence vs. contemporary gene flow: evolutionary history of the calcicole *Ranunculus alpestris* group (Ranunculaceae) in the European Alps and the Carpathians. Molecular Ecology 17: 4263–4275. 19378404 10.1111/j.1365-294x.2008.03908.x PMC2988486

[30] Schönswetter P, Tribsch A (2005) Vicariance and dispersal in the alpine perennial *Bupleurum stellatum* L. (Apiaceae). Taxon 54: 725–732.

[31] Poschlod P (2015) Geschichte der Kulturlandschaft. Stuttgart: Verlag Eugen Ulmer. 320 p.

[32] Poschlod P, Bonn S (1998) Changing dispersal processes in the Central European landscape since the last ice age: an explanation for the actual decrease of plant species richness in different habitats? Acta Botanica Neerlandica 47: 27–44.

[33] Bonn S, Poschlod P (1998) Ausbreitungsbiologie der Pflanzen Mitteleuropas: Grundlagen und kulturhistorische Aspekte. Wiesbaden: Quelle & Meyer Verlag. 404 p.

[34] Di Castri F (1989) History of biological invasions with special emphasis on the Old World In: Drake JA, Mooney HA, Di Castri F, Groves RH, Kruger FJ, Rejmánek M et al, editors. Biological invasions: a global perspective. Chichester, UK: Wiley & Sons pp. 1–30.

[35] Poschlod P, Abedi M, Bartelheimer M, Drobnik J, Rosbakh S, Saatkamp A (2013) Seed ecology and assembly rules in plant communities In: van der Maarel E, Franklin J, editors. Vegetation Ecology: John Wiley & Sons pp. 164–202.

[36] Hanotte O, Bradley DG, Ochieng JW, Verjee Y, Hill EW, Rege JEO (2002) African pastoralism: genetic imprints of origins and migrations. Science 296: 336–339. doi: 10.1126/science.1069878 11951043 10.1126/science.1069878

[37] Negrini R, Nijman IJ, Milanesi E, Moazami‐Goudarzi K, Williams JL, Erhardt G, et al (2007) Differentiation of European cattle by AFLP fingerprinting. Animal Genetics 38: 60–66. doi: 10.1111/j.1365-2052.2007.01554.x 17257190 10.1111/j.1365-2052.2007.01554.x

[38] Meindl C, Brune V, Listl D, Poschlod P, Reisch C (2016) Survival and postglacial immigration of the steppe plant *Scorzonera purpurea* to Central Europe. Plant Systematics and Evolution: 1–14.

[39] Abbott RJ, Comes HP (2004) Evolution in the Arctic: a phylogeographic analysis of the circumarctic plant, *Saxifraga oppositifolia* (Purple saxifrage). New Phytologist 161: 211–224.

[40] Alsos IG, Eidesen PB, Ehrich D, Skrede I, Westergaard K, Jacobsen GH, et al (2007) Frequent long-distance plant colonization in the changing Arctic. Science 316: 1606–1609. doi: 10.1126/science.1139178 17569861 10.1126/science.1139178

[41] Després L, Gielly L, Redoutet B, Taberlet P (2003) Using AFLP to resolve phylogenetic relationships in a morphologically diversified plant species complex when nuclear and chloroplast sequences fail to reveal variability. Molecular Phylogenetics and Evolution 27: 185–196. 12695084 10.1016/s1055-7903(02)00445-1

[42] Kropf M, Kadereit JW, Comes HP (2002) Late Quaternary distributional stasis in the submediterranean mountain plant *Anthyllis montana* L.(Fabaceae) inferred from ITS sequences and amplified fragment length polymorphism markers. Molecular Ecology 11: 447–463. 11918780 10.1046/j.1365-294x.2002.01446.x

[43] Kropf M (2008) Intraspecific patterns of European mountain plants: a morphometric analysis confirms molecular results in the submediterranean oreophyte *Anthyllis montana* L. (Fabaceae). Taxon 57: 511–524.

[44] Reisch C, Poschlod P, Wingender R (2003) Genetic differentiation among populations of *Sesleria albicans* Kit. ex Schultes (Poaceae) from ecologically different habitats in central Europe. Heredity 91: 519–527. doi: 10.1038/sj.hdy.6800350 14576746 10.1038/sj.hdy.6800350

[45] Reisch C, Poschlod P, Wingender R (2003) Genetic variation of *Saxifraga paniculata* Mill.(Saxifragaceae): molecular evidence for glacial relict endemism in central Europe. Biological Journal of the Linnean Society 80: 11–21.

[46] Reisch C (2008) Glacial history of *Saxifraga paniculata* (Saxifragaceae): molecular biogeography of a disjunct arctic‐alpine species from Europe and North America. Biological Journal of the Linnean Society 93: 385–398.

[47] Ronikier M, Costa A, Aguilar JF, Feliner GN, Küpfer P, Mirek Z (2008) Phylogeography of *Pulsatilla vernalis* (L.) Mill. (Ranunculaceae): chloroplast DNA reveals two evolutionary lineages across central Europe and Scandinavia. Journal of Biogeography 35: 1650–1664.

[48] Sanz M, Schönswetter P, Vallès J, Schneeweiss GM, Vilatersana R (2014) Southern isolation and northern long-distance dispersal shaped the phylogeography of the widespread, but highly disjunct, European high mountain plant *Artemisia eriantha* (Asteraceae). Botanical Journal of the Linnean Society 174: 214–226.

[49] Schönswetter P, Popp M, Brochmann C (2006) Rare arctic‐alpine plants of the European Alps have different immigration histories: the snow bed species *Minuartia biflora* and *Ranunculus pygmaeus*. Molecular Ecology 15: 709–720. doi: 10.1111/j.1365-294X.2006.02821.x 16499696 10.1111/j.1365-294X.2006.02821.x

[50] Skrede I, Eidesen PB, Portela RP, Brochmann C (2006) Refugia, differentiation and postglacial migration in arctic-alpine Eurasia, exemplified by the mountain avens (*Dryas octopetala* L.). Molecular Ecology 15: 1827–1840. doi: 10.1111/j.1365-294X.2006.02908.x 16689901 10.1111/j.1365-294X.2006.02908.x

[51] Bylebyl K, Poschlod P, Reisch C (2008) Genetic variation of *Eryngium campestre* L. (Apiaceae) in central Europe. Molecular Ecology 17: 3379–3388. doi: 10.1111/j.1365-294X.2008.03836.x 18564089 10.1111/j.1365-294X.2008.03836.x

[52] Meindl C (2012) New aspects in plant conservation-Phylogeography, population dynamics, genetics and management of steppe plants in Bavaria [Dissertation]. Regensburg: University of Regensburg. 139 p.

[53] Pérez-Collazos E, Sanchez-Gomez P, Jiménez JF, Catalán P (2009) The phylogeographical history of the Iberian steppe plant *Ferula loscosii* (Apiaceae): a test of the abundant‐centre hypothesis. Molecular Ecology 18: 848–861. doi: 10.1111/j.1365-294X.2008.04060.x 19207254 10.1111/j.1365-294X.2008.04060.x

[54] Polunin O (1980) Flowers of Greece and the Balkans: a field guide. Oxford: Oxford University Press. 592 p.

[55] Tutin TG (1980) Flora europaea. Cambridge: Cambridge University Press.

[56] Ellenberg H (1996) Vegetation Mitteleuropas mit den Alpen. Stuttgart: Ulmer. 1095 p.

[57] Smith GF, Figueiredo E (2010) *Sedum album*: a mainstay of European succulents. Cactus and Succulent Journal 82: 41–42.

[58] Brandes D (1995) The flora of old town centres in Europe. Urban Ecology as the Basis of Urban Planning: 49–58.

[59] Wittig R (2004) The origin and development of the urban flora of Central Europe. Urban Ecosystems 7: 323–329.

[60] Simkova K, Polesny Z (2015) Ethnobotanical review of wild edible plants used in the Czech Republic. Journal of Applied Botany and Food Quality 88: 49–67.

[61] Redzic S (2006) Wild edible plants and their traditional use in the human nutrition in Bosnia-Herzegovina. Ecology of Food and Nutrition 45: 189–232.

[62] Tardío J, Pardo de Santayana M, Morales R (2006) Ethnobotanical review of wild edible plants in Spain. Botanical Journal of the Linnean Society 152: 27–71.

[63] Özgen U, Kaya Y (2004) Ethnobotancal studies in the villages of the district of Ilica (Province Erzurum), Turkey. Economic Botany 58: 691–696.

[64] Cakilcioglu U, Turkoglu I (2010) An ethnobotanical survey of medicinal plants in Sivrice (Elazığ-Turkey). Journal of Ethnopharmacology 132: 165–175. doi: 10.1016/j.jep.2010.08.017 20713142 10.1016/j.jep.2010.08.017

[65] Meusel H, Jäger EJ, Weinert E, Rauschert S (1965) Vergleichende Chorologie der zentraleuropäischen Flora I–III. Jena: Gustav Fischer.

[66] Albers F (1998) Chromosomenzahlen der Farn- und Blütenpflanzen Deutschlands In: Wisskirchen R, Haeupler H, editors. Standardliste der Farn- und Blütenpflanzen Deutschlands. Stuttgart: Ulmer.

[67] t' Hart H (1991) Evolution and classification of the European *Sedum* species (Crassulaceae). Flora Mediterranea 2: 31–61.

[68] Dobes C, Vitek E (2000) Documented chromosome number checklist of Austrian vascular plants. Wien: Verlag des Naturhistorischen Museums Wien. 642 p.

[69] Rogers SO, Bendich AJ (1994) Extraction of total cellular DNA from plants, algae and fungi In: Gelvin S, Schilperoort RA, editors. Plant molecular biology manual. Boston: Kluwer Academic Publishers pp. 183–190.

[70] Vos P, Hogers R, Bleeker M, Reijans M, Van de Lee T, Hornes M, et al (1995) AFLP: a new technique for DNA fingerprinting. Nucleic Acids Research 23: 4407–4414. 7501463 10.1093/nar/23.21.4407 PMC307397

[71] Bonin A, Bellemain E, Bronken Eidesen P, Pompanon F, Brochmann C, Taberlet P (2004) How to track and assess genotyping errors in population genetics studies. Molecular Ecology 13: 3261–3273. doi: 10.1111/j.1365-294X.2004.02346.x 15487987 10.1111/j.1365-294X.2004.02346.x

[72] Parisod C, Christin PA (2008) Genome-wide association to fine-scale ecological heterogeneity within a continuous population of *Biscutella laevigata* (Brassicaceae). New Phytologist 178: 436–447. doi: 10.1111/j.1469-8137.2007.02361.x 18208468 10.1111/j.1469-8137.2007.02361.x

[73] Yeh FC, Yang RC, Boyle TBJ, Ye ZH, Mao JX (1997) POPGENE, the user-friendly shareware for population genetic analysis. University of Alberta, Canada: Molecular Biology and Biotechnology Centre.

[74] Peakall R, Smouse PE (2001) GenAlEx: genetic analysis in Excel. Population genetic software for teaching and research. 5 ed. Canberra, Australia: Australian National University. 10.1093/bioinformatics/bts460 PMC3463245 22820204

[75] Fischer M, Matthies D (1998) RAPD variation in relation to population size and plant fitness in rare *Gentianella germanica* (Gentianaceae). American Journal of Botany 85: 811–819. 21684965

[76] Slatkin M (1985) Rare alleles as indicators of gene flow. Evolution 39: 53–65. doi: 10.1111/j.1558-5646.1985.tb04079.x 28563643 10.1111/j.1558-5646.1985.tb04079.x

[77] Ehrich D (2006) AFLPdat: a collection of R functions for convenient handling of AFLP data. Molecular Ecology Notes 6: 603–604.

[78] Vekemans X (2002) AFLP-surv version 1.0. Bruxelles, Belgium: Distributed by the author. Laboratoire de Génétique et Ecologie Végétale, Université Libre de Bruxelles.

[79] Huson DH, Bryant D (2006) Application of phylogenetic networks in evolutionary studies. Molecular Biology and Evolution 23: 254–267. doi: 10.1093/molbev/msj030 16221896 10.1093/molbev/msj030

[80] Wright S (1965) The interpretation of population structure by F-statistics with special regard to systems of mating. Evolution 19: 395–420.

[81] Excoffier L, Smouse PE, Quattro JM (1992) Analysis of molecular variance inferred from metric distances among DNA haplotypes: application to human mitochondrial DNA restriction data. Genetics 131: 479–491. 1644282 10.1093/genetics/131.2.479 PMC1205020

[82] Mantel N (1967) The detection of disease clustering and a generalized regression approach. Cancer research 27: 209–220. 6018555

[83] Pritchard JK, Stephens M, Donnelly P (2000) Inference of population structure using multilocus genotype data. Genetics 155: 945–959. 10835412 10.1093/genetics/155.2.945 PMC1461096

[84] Evanno G, Regnaut S, Goudet J (2005) Detecting the number of clusters of individuals using the software STRUCTURE: a simulation study. Molecular Ecology 14: 2611–2620. doi: 10.1111/j.1365-294X.2005.02553.x 15969739 10.1111/j.1365-294X.2005.02553.x

[85] Earl DA, von Holdt BM (2012) STRUCTURE HARVESTER: a website and program for visualizing STRUCTURE output and implementing the Evanno method. Conservation Genetics Resources 4: 359–361.

[86] Jakobsson M, Rosenberg NA (2007) CLUMPP: a cluster matching and permutation program for dealing with label switvhing and multimodality in analysis of population structure. Bioinformatics 23: 1801–1806. doi: 10.1093/bioinformatics/btm233 17485429 10.1093/bioinformatics/btm233

[87] Rosenberg NA (2004) Distruct: a program for the graphical display of population structure. Molecular Ecology Notes 4: 137–138.

[88] Taberlet P, Gielly L, Pautou G, Bouvet J (1991) Universal primers for amplification of three non-coding regions of chloroplast DNA. Plant molecular biology 17: 1105–1109. 1932684 10.1007/BF00037152

[89] Hamilton MB (1999) Four primer pairs for the amplification of chloroplast intergenic regions with intraspecific variation. Molecular Ecology 8: 521–523. 10199016

[90] Shaw J, Lickey EB, Schilling EE, Small RL (2007) Comparison of whole chloroplast genome sequences to choose noncoding regions for phylogenetic studies in angiosperms: the tortoise and the hare III. American Journal of Botany 94: 275–288. doi: 10.3732/ajb.94.3.275 21636401 10.3732/ajb.94.3.275

[91] Altschul SF, Boguski MS, Gish W, Wootton JC (1994) Issues in searching molecular sequence databases. Nature Genetics 6: 119–129. doi: 10.1038/ng0294-119 8162065 10.1038/ng0294-119

[92] McWilliam H, Li W, Uludag M, Squizzato S, Park YM, Buso N, et al (2013) Analysis tool web services from the EMBL-EBI. Nucleic Acids Research 41: W597–W600. doi: 10.1093/nar/gkt376 23671338 10.1093/nar/gkt376 PMC3692137

[93] Hall TA (1999) BioEdit: a user-friendly biological sequence alignment editor and analysis program for Windows 95/98/NT. Nucleic Acid Research 41: 95–98.

[94] Clement M, Posada D, Crandall KA (2000) TCS: a computer program to estimate gene genealogies. Molecular Ecology 9: 1657–1659. 11050560 10.1046/j.1365-294x.2000.01020.x

[95] van Ham RCHJ, 't Hart H (1998) Phylogenetic relationships in the Crassulaceae inferred from chloroplast DNA restriction-site variation. American Journal of Botany 85: 123–134. 21684886

[96] Swofford D (2002) PAUP*. Phylogenetic analysis using parsimony (*and other methods). Sunderland, Masschusetts: Sinnauer Associates.

[97] Weising K, Gardner RC (1999) A set of conserved PCR primers for the analysis of simple sequence repeat polymorphisms in chloroplast genomes of dicotyledonous angiosperms. Genome 42: 9–19. 10207998

[98] Gaudeul M, Till-Bottraud I, Barjon F, Manel S (2004) Genetic diversity and differentiation in *Eryngium alpinum* L. (Apiaceae): comparison of AFLP and microsatellite markers. Heredity 92: 508–518. doi: 10.1038/sj.hdy.6800443 15014426 10.1038/sj.hdy.6800443

[99] Feliner GN (2011) Southern European glacial refugia: a tale of tales. Taxon 60: 365–372.

[100] Leipold M, Tausch S, Poschlod P, Reisch C (2017) Species distribution modeling and molecular markers suggest longitudinal range shifts and cryptic northern refugia of the typical calcareous grassland species *Hippocrepis comosa* (horseshoe vetch). Ecology and Evolution 7: 1919–1935. doi: 10.1002/ece3.2811 28331599 10.1002/ece3.2811 PMC5355195

[101] Tausch S, Leipold M, Poschlod P, Reisch C (2017) Molecular markers provide evidence for a broad fronted recolonization of the widespread calcareous grassland species *Sanguisorba minor* (Salad Burnett) from southern and cryptic northern refugia. Plant Biology: in press. 10.1111/plb.12570 28387987

[102] Barton NH, Hewitt GM (1985) Analysis of hybrid zones. Annual Review of Ecology and Systematics 16: 113–148.

[103] Flanagan NS, Mason PL, Gosalvez J, Hewitt GM (1999) Chromosomal differentiation through an Alpine hybrid zone in the grasshopper *Chorthippus parallelus*. Journal of Evolutionary Biology 12: 577–585.

[104] Hewitt GM (1975) A sex-chromosome hybrid zone in the grasshopper *Podisma pedestris* (Orthoptera: Acrididae). Heredity 35: 375–387. 1061710 10.1038/hdy.1975.108

[105] Lugon-Moulin N, Brünner H, Wyttenbach A, Hausser J, Goudet J (1999) Hierarchical analyses of genetic differentiation in a hybrid zone of *Sorex araneus* (Insectivora: Soricidae). Molecular Ecology 8: 419–431.

[106] Médail F, Diadema K (2009) Glacial refugia influence plant diversity patterns in the Mediterranean Basin. Journal of Biogeography 36: 1333–1345.

[107] Vernesi C, Pecchioli E, Caramelli D, Tiedemann R, Randi E, Bertorelle G (2002) The genetic structure of natural and reintroduced roe deer (*Capreolus capreolus*) populations in the Alps and central Italy, with reference to the mitochondrial DNA phylogeography of Europe. Molecular Ecology 11: 1285–1297. 12144651 10.1046/j.1365-294x.2002.01534.x

[108] Koch M, Bernhardt KG (2004) Comparative biogeography of different cytotypes of the European annual *Microthlaspi perfoliatum* (Brassicaceae): refugia, diversity centres and post glacial colonization. American Journal of Botany 91: 115–124. doi: 10.3732/ajb.91.1.115 21653368 10.3732/ajb.91.1.115

