# Supplementary figures and images for: Phylogeography of a tough rock survivor in European dry grasslands
Source: PLoS One. 2017 Jun 22;12(6):e0179961. doi: 10.1371/journal.pone.0179961 (PMC5481016; doi:10.1371/journal.pone.0179961)

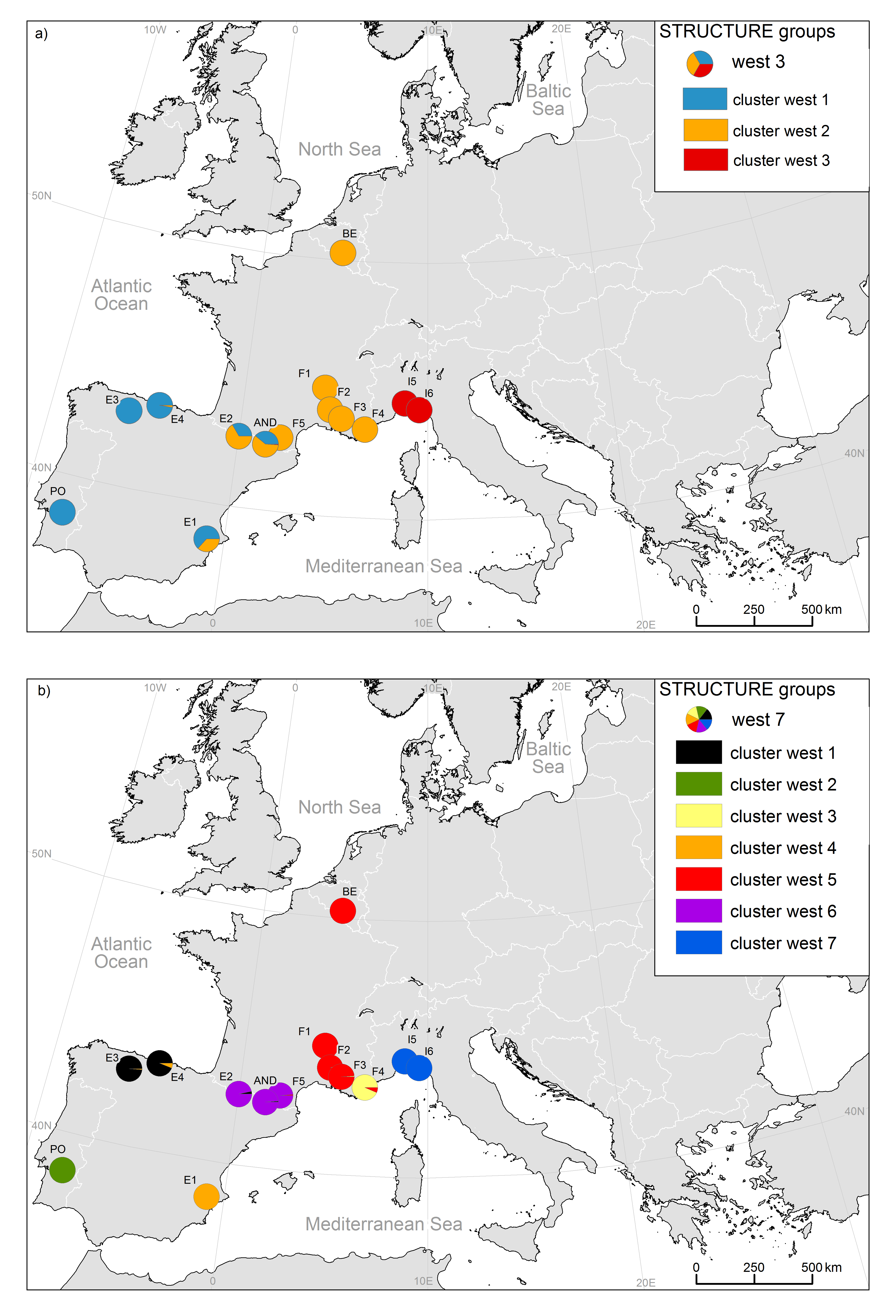

Supplement: S1 Fig — Most probable subdivision of the western group was a) K = 3 and b) K = 7. (TIF) [file pone.0179961.s001.tif]

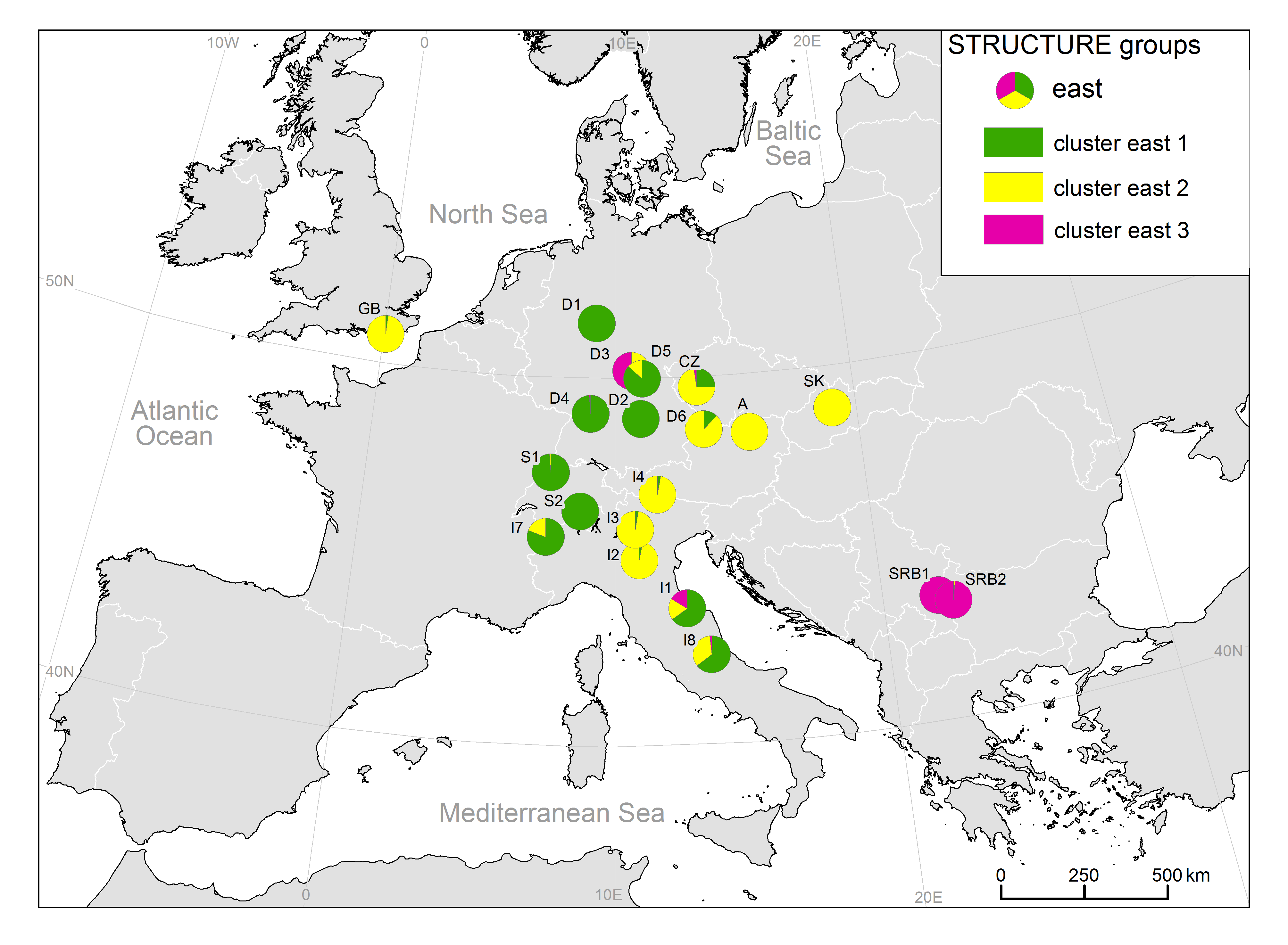

Supplement: S2 Fig — Most probable subdivision of the eastern group was K = 3. (TIF) [file pone.0179961.s002.tif]
