# Supplementary material for: Phylogeography of a tough rock survivor in European dry grasslands
Source: PLoS One. 2017 Jun 22;12(6):e0179961. doi: 10.1371/journal.pone.0179961 (PMC5481016; doi:10.1371/journal.pone.0179961)
Supplement: S2 Table — Concatenated sequences of S. album individuals from 32 populations that were used for all further analyses of chloroplast sequences. (DOCX) [file pone.0179961.s004.docx]

*PLOS ONE*

**SUPPORTING INFORMATION**

**Phylogeography of a tough rock survivor in European dry grasslands**

Daniela Listl*, Peter Poschlod and Christoph Reisch

**Appendix S1**

**Table S1**. AFLP matrix of 385 individuals from 34 populations of *S. album*.

**Table S2**. Polymorphic sites of trnL-trnF chloroplast regions based on concatenated sequences of *S. album* individuals from 32 populations.

|  | ***tab f*** | | | | | | | | | | | | | | | | ***tab c*** | | | | | | | | | | | | | |
| --- | --- | --- | --- | --- | --- | --- | --- | --- | --- | --- | --- | --- | --- | --- | --- | --- | --- | --- | --- | --- | --- | --- | --- | --- | --- | --- | --- | --- | --- | --- |
| **name** | ***46*** | ***107*** | ***160*** | ***217*** | ***238*** | ***251-252*** | ***282*** | ***290*** | ***330-331*** | ***311*** | ***339-350*** | ***335*** | ***378*** | ***374*** | ***382*** | ***447*** | ***467*** | ***504*** | ***539*** | ***561-562*** | ***578*** | ***585 -594*** | ***599-600*** | ***604*** | ***626-628*** | ***637*** | ***647-648*** | ***686*** | ***742*** | ***767*** |
| **A** | T | G | T | C | T | AT | T | T | C | CT | - | - | A | T | G | a | T | T | A | CG | A | *^4^ | -- | A | GTA | G | CA | A | T | A |
| **AND** | T | G | T | C | A | -- | T | T | C | CT | - | - | A | T | G | a | T | T | A | CG | A | *^4^ | -- | A | GTA | T | Cg | A | T | A |
| **BE** | T | G | T | C | A | AT | T | T | C | CT | - | - | A | T | G | a | T | T | A | CG | A | *^4^ | -- | A | GTA | G | CG | A | T | A |
| **CZ** | T | G | T | C | T | AT | T | T | C | CT | - | - | A | T | G | a | T | T | A | CG | A | *4 | -- | A | GTA | G | CA | A | T | A |
| **D1** | T | G | T | C | T | AT | T | T | C | CT | - | - | A | T | G | a | T | T | A | CG | A | *4 | -- | A | TAC | G | Ca | A | T | A |
| **D2** | T | G | T | C | T | AT | T | T | C | CT | - | - | A | T | G | a | T | T | A | CG | A | *4 | -- | A | GTA | G | CA | A | T | A |
| **D3** | G | A | T | T | A | -- | T | T | C | -- | T | *1 | C | T | G | a | T | T | - | TT | - | *4 | -- | C | GTA | G | AG | A | T | A |
| **D4** | T | G | T | C | T | AT | T | T | C | CT | - | - | A | T | G | a | T | T | A | CG | A | *4 | -- | A | GTA | G | CA | A | T | A |
| **D5** | T | G | T | C | T | AT | T | T | C | CT | - | - | A | T | G | a | T | T | A | CG | A | *4 | -- | A | GTA | G | CA | C | T | A |
| **D6** | T | G | T | C | T | AT | T | T | C | CT | - | - | A | T | G | a | T | T | A | CG | A | *4 | -- | A | GTA | G | CA | A | T | A |
| **E1** | T | G | T | C | A | -- | T | T | C | CT | - | - | A | T | G | a | T | T | A | CG | A | *4 | -- | A | GTA | T | Cg | A | G | A |
| **E2** | T | G | T | C | A | AT | T | T | C | CT | - | - | A | T | G | a | T | T | A | CG | A | *4 | -- | A | GTA | G | Cg | A | T | A |
| **E3** | T | G | T | C | A | -- | T | T | C | CT | - | - | A | T | G | a | T | T | A | CG | A | *4 | -- | A | GTA | T | Cg | A | T | A |
| **E4** | T | G | T | C | T | AT | T | T | C | CT | - | - | A | T | G | A | T | T | A | CG | A | *4 | -- | A | GTA | G | Ca | A | G | A |
| **F1** | T | G | T | C | A | AT | A | T | C | CT | - | - | A | T | G | a | T | T | A | CG | A | *4 | -- | A | GTA | G | CG | A | T | A |
| **F2** | T | G | T | C | A | AT | A | A | C | CT | - | - | A | A | G | a | T | T | A | CG | A | *4 | -- | A | GTA | G | Cg | A | T | A |
| **F3** | T | G | T | C | A | AT | T | T | C | CT | - | - | A | T | G | a | G | T | A | CG | A | *4 | -- | A | GTA | G | CG | A | T | A |
| **F4** | T | G | T | C | A | AT | T | T | C | CT | - | - | A | T | G | a | T | T | A | CG | A | *4 | -- | A | GTA | G | CG | A | T | A |
| **I1** | T | G | T | C | T | AT | T | T | C | CT | - | - | A | T | G | a | T | A | A | CG | - | *4 | -- | A | GTA | G | CA | A | T | A |
| **I2** | T | G | T | C | T | AT | T | T | C | CT | - | - | A | T | G | a | T | C | A | CG | A | *4 | -- | A | GTA | G | CA | A | T | A |
| **I3** | T | G | T | C | T | AT | T | T | C | CT | - | - | A | T | G | a | T | T | A | CG | A | *4 | -- | A | GTA | G | CA | A | T | A |
| **I4** | T | G | T | C | T | AT | T | T | C | CT | - | - | A | T | G | a | T | T | A | CG | A | *4 | -- | A | GTA | G | CA | A | T | A |
| **I5** | G | G | T | C | A | -- | T | T | C | -- | - | ^*2^ | C | T | G | a | T | T | - | TT | - | -------- | -- | C | GTA | G | AG | A | T | A |
| **I6** | T | G | T | C | A | -- | T | T | C | CT | - | - | A | T | G | a | T | T | A | CG | A | *4 | -- | A | GTA | T | CG | A | T | A |
| **I7** | T | G | T | C | T | AT | T | T | C | CT | - | - | A | T | G | a | T | T | A | CG | A | *4 | -- | A | GTA | G | C- | A | T | C |
| **I8** | T | G | T | C | A | -- | T | T | C | CT | - | - | A | T | G | a | T | T | A | CG | A | *4 | -- | A | GTA | T | Cg | A | T | A |
| **PO** | T | G | T | C | A | -- | T | T | C | CT | - | ^*3^ | A | T | G | a | T | T | A | CG | A | *4 | -- | A | GTA | T | CG | A | T | A |
| **S1** | T | G | A | C | T | AT | T | T | C | CT | - | - | A | T | G | a | T | T | A | CG | A | *4 | -- | A | GTA | G | CC | A | T | A |
| **S2** | T | G | T | C | T | AT | T | T | C | CT | - | - | A | T | T | a | T | T | A | CG | A | *4 | -- | A | GTA | G | CA | A | T | A |
| **SK** | T | G | T | C | T | AT | T | T | C | CT | - | - | A | T | G | a | t | T | A | CG | A | *4 | -- | A | GTA | G | CA | A | T | A |
| **SRB1** | G | A | T | T | A | -- | T | T | A | -- | T | *1 | C | T | G | a | T | T | - | TT | A | *4 | AT | C | GTA | G | AG | A | T | A |
| **SRB2** | G | A | T | T | A | -- | T | T | A | -- | T | *1 | C | T | G | a | T | T | - | TT | A | *4 | AT | C | GTA | G | AG | A | T | A |
|  |  |  |  |  |  |  |  |  |  |  |  |  |  |  |  |  |  |  |  |  |  |  |  |  |  |  |  |  |  |  |

*^1^  ATAATATATTAA, *^2^  A-------TTAA, *^3^  AAAAT-TATTAT, *^4^ AAGTTTGT
